# Examining patient-reported late toxicity and its association with quality of life and unmet need for symptom management among nasopharyngeal cancer survivors: a cross-sectional survey

**DOI:** 10.3389/fonc.2024.1378973

**Published:** 2024-04-17

**Authors:** Victor C. W. Tam, Jerry C. F. Ching, Sindy S. T. Yip, Virginia H. Y. Kwong, Catherine P. L. Chan, Kenneth C. W. Wong, Shara W. Y. Lee

**Affiliations:** ^1^ Department of Health Technology and Informatics, Faculty of Health and Social Sciences, The Hong Kong Polytechnic University, Hong Kong, Hong Kong SAR, China; ^2^ Department of Clinical Oncology, Prince of Wales Hospital, Hong Kong, Hong Kong SAR, China; ^3^ Department of Otorhinolaryngology, Head and Neck Surgery, Faculty of Medicine, The Chinese University of Hong Kong, Hong Kong, Hong Kong SAR, China

**Keywords:** nasopharyngeal cancer, survivorship, radiation therapy, late toxicities, symptom burden, unmet need, patient-reported outcomes, health-related quality of life

## Abstract

**Introduction:**

Alongside the improved survival of nasopharyngeal cancer (NPC), late radiation toxicities are alarmingly hampering survivors’ quality of life. A patient-reported symptom burden survey is lacking to address the unmet need for symptom management among local NPC survivors.

**Methods:**

A single-center cross-sectional survey was conducted on 211 NPC survivors who had completed radiation therapy for three to 120 months. We employed the Chinese version M. D. Anderson Symptom Inventory - Head & Neck Module (MDASI-HN-C), Functional Assessment of Cancer Therapy - Head & Neck (FACT-HN-C), and a question extracted from the Cancer Survivors’ Unmet Needs Measure (CaSUN).

**Results:**

Two hundred valid responses were collected. Participants suffered from at least four moderate to severe symptoms (mean = 4.84, SD = 4.99). The top five severe symptoms were dry mouth, mucus problems, difficulty swallowing or chewing, teeth or gum problems, and memory problems. MDASI-HN-C subscales were negatively correlated with the physical, emotional, functional, and HN-specific domains of the FACT-HN-C. The unmet need for symptom management was positively associated with symptom burden, either general symptoms (Adjusted odds ratio [OR_adj_] = 1.566, 95% CI = 1.282 – 1.914, p < 0.001) or top-5 symptoms (OR_adj_ = 1.379, 95% CI = 1.185 – 1.604, p < 0.001), while negatively associated with post-RT time (OR_adj_ = 0.981, 95% CI [0.972, 0.991], p < 0.001).

**Conclusion:**

Virtually all NPC survivors suffer from late toxicities, which interplay with survivors’ perceptions intricately to affect their unmet needs for symptom management. Personalized supportive care strategies with regular assessments and stratifications are warranted.

## 1 Introduction

Nasopharyngeal cancer (NPC) is a prevailing head and neck (H&N) cancer in Southeast Asia, including Hong Kong. Technological advancements in radiation therapy (RT) with neoadjuvant and concurrent chemotherapy have significantly improved nasopharyngeal cancer (NPC) patients’ survival in the last decade (1). While NPC survivors are expected to live longer after treatment completion, they are grappling with long-term toxicities as a life-long challenge after the acute side effects subsided (2). For effective management of late toxicity in supportive care, it is crucial to comprehensively understand the prevalence and severity of each toxicity, along with the overall symptom burden.

The prevalence and severity of late treatment toxicities were investigated in numerous studies, focusing on various emerging side effects (2–5). However, the figures reported were considerably different among studies. For example, Fong et al. (6) revealed a widely varied prevalence of dysphagia from 13.0% to 93.5% in a scoping review covering 28 studies. This has been attributed to the heterogeneity of assessment tools and patients’ characteristics such as age, tumor staging and treatment techniques.

Two recently published retrospective studies have attempted to examine several major clinician-reported late toxicities among Chinese NPC survivors up to 10 to 15 years follow-up, including hearing impairment, xerostomia, skin problems, and hypothyroidism (3, 4). Surprisingly, the prevalence and severity varied significantly despite both studies employing the Radiation Therapy Oncology Group (RTOG) grading system. Clinician-reported outcomes have been challenged with its rater dependency, in which clinicians may unintentionally underreport toxicities (7, 8). There has been a growing awareness of incorporating patient-reported outcomes (PRO) to complement the assessment of toxicity among cancer patients and survivors (9). Yet, a comprehensive survey on PRO regarding toxicities in NPC survivors is lacking. Though toxicities were conceptually innate to health-related quality of life (HRQoL), HRQoL measures multidimensional facets in which each domain may not interact with toxicities likewise. Therefore, this study aims to examine the subjective symptom burden, quality of life, and unmet need for symptom management among local NPC survivors.

## 2 Methods

### 2.1 Study design and setting

This cross-sectional survey recruited 211 local NPC survivors from the Department of Clinical Oncology and the Department of Otorhinolaryngology, Head and Neck Surgery of the Prince of Wales Hospital between December 13, 2022, and April 18, 2023. A convenience sampling strategy was applied. Ethics approval was obtained from the Institutional Review Board of The Hong Kong Polytechnic University (Reference number: HSEARS20220321007) and the Clinical Research Ethics Committee of the Joint Chinese University of Hong Kong-New Territories East Cluster (Reference number: 2022.472).

### 2.2 Study population & sample size calculation

Chinese NPC survivors who have completed primary radiation therapy for three to 120 months were recruited at the follow-up clinic from the Department of Clinical Oncology and the Department of Otorhinolaryngology, Head and Neck Surgery of the Prince of Wales Hospital. Inclusion criteria were (i) Chinese NPC survivors who completed primary radiation therapy for three to 120 months, (ii) were able to read and understand Chinese, and (iii) to give informed consent.

There were 8,345 new NPC cases between 2010 and 2019 in Hong Kong. Assuming similar incidences in 2020 and 2021, we estimated that 8,345 NPC survivors have completed their primary treatment for three to 120 months as of 2022. Using Epi Info™ (Centers for Disease Control and Prevention, CDC US), we calculated that a sample size of 189 is required with a 5% margin of error and a 97% confidence level. The calculation was based on the prevalence of dysphagia of 11.6%, a primary concerning symptom among NPC survivors (10). A final sample size of 210 was estimated to account for potential dropouts during data collection.

### 2.3 Instruments

A survey consisting of two standard questionnaires and one single question was employed anonymously: (i) The M. D. Anderson Symptom Inventory - Head & Neck Module - Chinese (MDASI-HN-C); (ii) the Functional Assessment of Cancer Therapy - Head & Neck - Chinese (FACT-HN-C) and (iii) a single question regarding the unmet need for symptom management extracted from the Cancer Survivors’ Unmet Needs Measure (CaSUN). A sample questionnaire in English is attached in **Appendix 1**.

The MDASI-HN-C consists of 28 items, including 22 severity questions for physical and psychological symptoms, as well as six symptom interference items (11). All items are rated on an 11-point Likert scale, i.e., 0-10, to measure symptom severity or interference over the last 24 hours. Scoring five or above was defined as a moderate to severe symptom based on previous literature (12, 13). Three subscales were identified in the initial development process: core symptoms (13 items), HN-specific symptoms (9 items) and symptom interference (6 items). According to The MDASI User Guide (14), the mean score of the most severe symptom items could be calculated to represent symptom burden. The MDASI-HN-C was validated in an NPC patient’s cohort and demonstrated to have excellent internal consistency, with Cronbach’s α ranging from 0.85-0.91 for all three subscales (15). Besides, the two-factor structures of the core and HN-specific symptoms were revealed in NPC patients to illustrate their symptom severity, comprising a total of four factors, including general, gastrointestinal, nutrition impact and social interaction impact symptoms.

The FACT-HN-C version 4 contains 39 items, which are further divided into physical (7 items), social/family (7 items), emotional (6 items), functional (7 items) well-being, and HN disease-specific concerns (12 items). A 5-point Likert scale was employed for each question. The FACT-HN-C was demonstrated with a good internal consistency of Cronbach’s α of 0.87 in an NPC cohort (16). This scale has been reported to be user-friendly, with a completion time of 10 minutes. It is considered to be an all-rounded questionnaire for cancer patients and survivors (17, 18).

The need for symptom management was assessed with a single question from The Cancer Survivors’ Unmet Needs Measure (CaSUN): ‘In the last month, did you need help managing ongoing side effects and/or complications of treatment?’ Respondents selected from five options: (i) no need/not applicable, (ii) need met, (iii) weak unmet need, (iv) moderate unmet need, and (v) strong unmet need. Responses indicating a weak, moderate, or strong unmet need (iii, iv, and v) were collectively considered evidence of an unmet need for symptom management.

### 2.4 Data collection

Eligible patients were shortlisted by an on-site research nurse before recruitment. The research objectives and procedures were explained to each participant before obtaining their consent. Participants were asked to complete the whole set of questionnaires mainly by themselves, with the researcher readily available to provide assistance. No personally identifiable information was collected.

### 2.5 Statistical analysis

Data were analyzed using IBM SPSS Statistics version 26. Descriptive statistics were used to give an overview of the patient’s characteristics and symptom scores. Symptom severity and distress were calculated according to the 4-factor model suggested by Xiao et al. (15). The mean score of the top-5 items (MeanTop5) was calculated as an alternative scale to represent symptom burden according to the MDASI User Guide.

Correlations between each MDASI symptom and post-treatment duration were assessed using Spearman’s correlation coefficient (ρ). Post-treatment duration was categorized into ten intervals. Kruskal-Wallis test was employed to compare the severity of each MDASI symptom across these different post-treatment time intervals. Correlations between each MDASI-HN-C subscales and FACT-HN-C domains were assessed using Pearson’s correlation (r). Associations between each MDASI subscale and each patient’s characteristics were assessed using univariate linear regression to identify significant predictors. Univariate logistic regression (ULR) was used to identify associated MDASI subscales and patients’ characteristics for the unmet need for symptom management.

Multiple logistic regression (MLR) was used to establish predictive models for the unmet need for symptom management, with significantly associated patients’ characteristics in the ULR entered into the first block of the MLR, followed by the associated MDASI subscales. Backward selection was conducted until all remaining variables were significant. Two predictive MLR models were established based on the 4-factor model (15) and the MeanTop5 scale. A p-value < 0.05 was considered statistically significant. Open-ended questions were analyzed using thematic analysis to identify additional symptoms and concerns.

## 3 Results

We approached 257 eligible NPC survivors, and 211 of them consented and participated in the study (response rate = 82.1%). Two hundred valid responses were collected and included in the final analysis after removing incomplete responses due to substantial missing data. The median follow-up duration was 46 months (Interquartile range = 23 - 83). The demographics and disease characteristics are summarized in **Tables 1**, **2** respectively.

**Table 1:** Demographic characteristics.

Demographic characteristic (N=200)	Median (IQR)
Age	60 (51-65)
Age at diagnosis	54 (46-61)
	Frequency (Percentage [%])
Gender	
Female	52 (26.0)
Male	148 (74.0)
Educational level	
Not at all	5 (2.5)
Primary school	123 (61.5)
Secondary school	33 (16.5)
Diploma and bachelor’s degree	26 (13.0)
Master’s degree or above	13 (6.5)
Employment status	
Full-time job	81 (40.5)
Part-time job	19 (9.5)
Sick leave	2 (1.0)
Not employed	14 (7.0)
Retired	74 (37.0)
Housewife	9 (4.5)
Student	1 (0.5)
Marital status	
Single	24 (12.0)
In a relationship	2 (1.0)
Married	160 (80.0)
Divorced	9 (4.5)
Widowed	4 (2.0)
Not to mention	1 (0.5)
Living status	
Living alone	10 (5.0)
Living with spouse	52 (26.0)
Living with family members	132 (66.0)
Living with others	2 (1.0)
Not to mention	2 (1.0)
Others	2 (1.0)
Current smoker	
Yes	16 (8.0)
No	184 (92.0)
Current drinker	
Yes	56 (28.0)
No	144 (72.0)

IQR, Interquartile range.

**Table 2:** Disease and treatment characteristics.

Characteristic (N=200)	Median (IQR)	
Post-treatment duration (Month)	46 (23-83)
	Frequency (Percentage [%])	
Post-treatment time intervals (Month)		
3-12	19 (9.5)
13-24	34 (17.0)
25-36	30 (15.0)
37-48	21 (10.5)
49-60	19 (9.5)
61-72	13 (6.5)
73-84	17 (8.5)
85-96	18 (9.0)
97-108	18 (9.0)
109-120	11 (5.5)
Received chemotherapy		
Yes	151 (75.5)
No	49 (24.5)
T-stage ^a^		
1	94 (47.0)
2	22 (11.0)
3	65 (32.5)
4	19 (9.5)
N-stage ^a^		
0	55 (27.5)
1	56 (28.0)
2	62 (31.0)
3	26 (13.0)
Missing	1 (0.5)
M-stage ^a^		
0	196 (98.0)
1	3 (1.5)
Missing	1 (0.5)
TNM stage		
1	39 (19.5)
2	35 (17.5)
3	84 (42.0)
4	42 (21.0)
Number of chronic illnesses		
0	116 (58.0)
1	54 (27.0)
2	19 (9.5)
3	10 (5.0)
4	1 (0.5)
Disease (N=200)	Frequency	Prevalence (%)
Heart disease	4	2.0
Diabetes mellitus	19	9.5
Stroke	5	2.5
Asthma	2	1.0
Hypercholesterolemia	31	15.5
Kidney disease	4	2.0
Hypertension	61	30.5
Chronic Pulmonary Disease	0	0

IQR, Interquartile range.

a The 8th edition of the International Union against Cancer/American Joint Committee on Cancer (UICC/AJCC) staging system for nasopharyngeal carcinoma was used.

### 3.1 Self-reported symptom burden

The mean (*M*) and standard deviations (SD) of the total MDASI symptom severity scores were 48.75 ± 36.24, with a possible maximum total score of 220. The majority of participants (99.0%) suffered from at least one symptom. On average, participants suffered from 4.84 (SD = 4.99) moderate to severe symptoms. There were 152 respondents (76.0%) suffering from at least one moderate to severe symptom.

Descriptive statistics of each symptom and interference item were summarized in **Table 3**. The top-5 scored symptoms were dry mouth (*M* = 5.47), problem with mucus in the mouth and throat (*M* = 4.27), difficulty swallowing or chewing (*M* = 3.38), problem with teeth or gums (*M* = 2.98) and problem with remembering things (*M* = 2.94). In addition, dry mouth (63.5%), problems with mucus in the mouth and throat (44.5%), difficulty swallowing or chewing (36.0%) and problems with teeth or gums (31.0%) were also the four most common symptoms rated moderate to severe.

**Table 3:** Descriptive statistics of each symptom.

Symptom (N=200)	Scores^a^ (Mean ± SD)	Prevalence of symptoms (%)^b^	Prevalence of moderate to severe symptoms (%)^c^
Dry mouth	5.47 ± 2.90	96.5	63.5
Problem with mucus in your mouth and throat	4.27 ± 3.17	84.0	44.5
Difficulty swallowing or chewing	3.38 ± 3.02	77.5	36.0
Problem with your teeth or gums	2.98 ± 3.17	63.5	31.0
Problem with remembering things	2.94 ± 2.63	75.5	26.0
Problem with tasting food	2.77 ± 2.80	67.0	26.5
Fatigue (tiredness)	2.56 ± 2.64	63.0	26.5
Disturbed sleep	2.44 ± 2.68	59.0	26.5
Drowsy (sleepy)	2.41 ± 2.50	64.5	23.0
Distressed (upset)	2.29 ± 2.66	58.0	22.2
Choking or coughing	2.11 ± 2.65	59.0	15.5
Numbness or tingling	1.94 ± 2.67	50.8	17.1
Lack of appetite	1.74 ± 2.37	50.5	16.0
Constipation	1.66 ± 2.47	45.0	15.5
Sad	1.64 ± 2.35	44.0	17.5
Pain	1.61 ± 2.38	43.0	15.5
Difficulty with voice or speech	1.49 ± 2.22	44.2	12.6
Shortness of breath	1.48 ± 2.19	42.5	13.0
Mouth or throat sores	1.46 ± 2.30	42.5	14.5
Skin pain, burning or rash	1.01 ± 2.06	28.5	10.0
Nausea	0.65 ± 1.76	19.5	6.0
Vomiting	0.50 ± 1.51	15.5	5.5
Interference item (N=200)	Scores (Mean ± SD)	Prevalence of symptoms (%)^a^	Prevalence of moderate to severe symptoms (%)^b^
General activity	2.00 ± 2.52	53.5	20.0
Mood	1.84 ± 2.47	49.5	16.5
Work (including work around the house)	2.11 ± 2.71	50.3	20.6
Relations with other people	1.24 ± 2.17	33.5	13.5
Walking	1.81 ± 2.55	45.5	16.5
Enjoyment of life	1.94 ± 2.73	48.5	20.5

a Sort in descending order of MDASI mean score.

b MDASI scored 1 or above.

c MDASI scored 5 or above.

SD, standard deviation.

**Figure 1:**
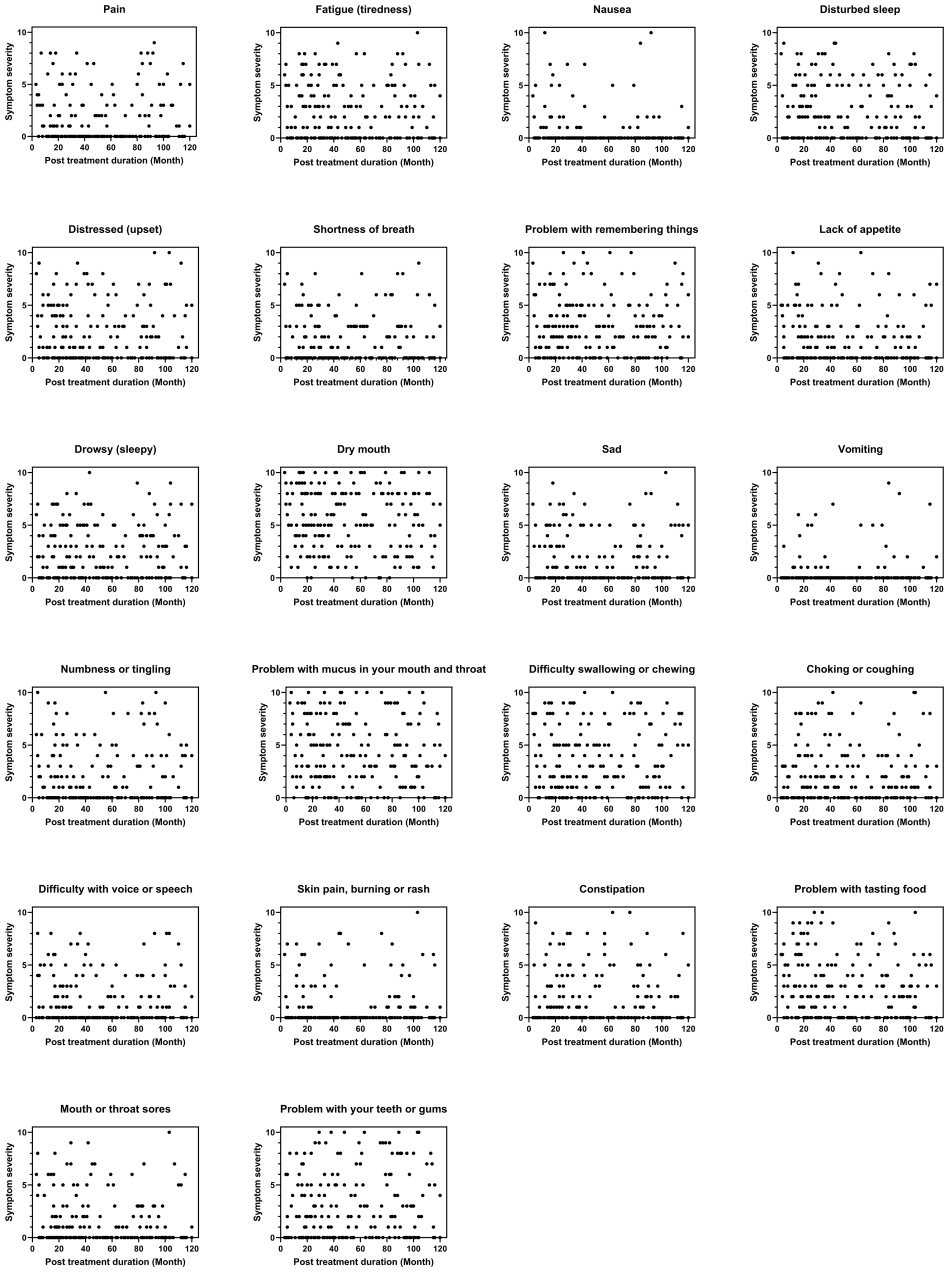
Symptom severity across post-treatment duration (N = 200). Figures were constructed using GraphPad Prism version 9 (GraphPad Software, San Diego, USA). A dispersed pattern of the severity of each symptom across post treatment duration was shown.

**Figure 1** illustrates the dispersed pattern of symptom severity across post-treatment duration. There was a significant correlation between post-treatment duration and the severity of nausea (ρ[198] = -0.141, p = 0.047), lack of appetite (ρ[198] = -0.150, p = 0.034), and dysgeusia (ρ[198] = -0.154, p = 0.029). However, the Kruskal-Wallis test did not reveal any significant differences in the severity of MDASI symptoms across different post-treatment time intervals.

The mean score of each MDASI symptom severity subscales were general (*M* = 2.41, SD = 1.82), gastrointestinal (*M* = 0.58, SD = 1.51), nutrition impact (*M* = 2.75, SD = 2.01) and social interaction impact symptoms (*M* = 1.54, SD = 1.83). The mean score of six interference items was computed to represent symptom distress (*M* = 1.82, SD = 2.11), and the mean score of the top-5 items (MeanTop5) was calculated as an alternative scale to represent symptom burden (*M* = 3.81, SD = 2.25).

### 3.2 MDASI-HN-C subscales

Most MDASI-HN-C subscales were negatively correlated with the physical, emotional, functional, HN-specific domains and the total score of the FACT-HN-C scale. However, the social/family domain was only associated with the social interaction impact symptoms among all MDASI subscales (r = -.139, p = 0.049). **Table 4** summarizes the correlations between MDASI-HN-C subscales and FACT-HN-C scale domains.

**Table 4:** Pearson correlation coefficients (r) between MDASI subscales and FACT-HN-C domains.

MDASI-HN-C subscale	FACT-HN-C domain					FACT-HN-C Total
	Physical	Social/family	Emotional	Functional	HN-specific	
General	-.747^‡^	-.018	-.624^‡^	-.361^‡^	-.597^‡^	-.566^‡^
Gastrointestinal	-.307^‡^	.055	-.304^‡^	-.126	-.265^‡^	-.223^†^
Nutrition impact	-.641^‡^	-.060	-.501^‡^	-.263^‡^	-.556^‡^	-.492^‡^
Social interaction impact	-.622^‡^	-.139^*^	-.503^‡^	-.338^‡^	-.539^‡^	-.527^‡^
Interference	-.787^‡^	-.098	-.625^‡^	-.390^‡^	-.616^‡^	-.612^‡^
MeanTop5	-.643^‡^	-.050	-.507^‡^	-.253^‡^	-.550^‡^	-.486^‡^

^*^p<0.05.

^†^p<0.01.

^‡^p<0.001.

Associations between the MDASI subscale and patients’ characteristics were summarized in **Supplementary Table 1**. None of the MDASI subscales, i.e., four severity and one interference scales, nor the MeanTop5 were associated with post-RT months, patient’s age or age at diagnosis. The tumor stage under the TNM staging system was associated with the general, gastrointestinal and nutrition impact symptoms, as well as the interference and the MeanTop5 items.

### 3.3 Unmet need for symptom management

**Figure 2:**
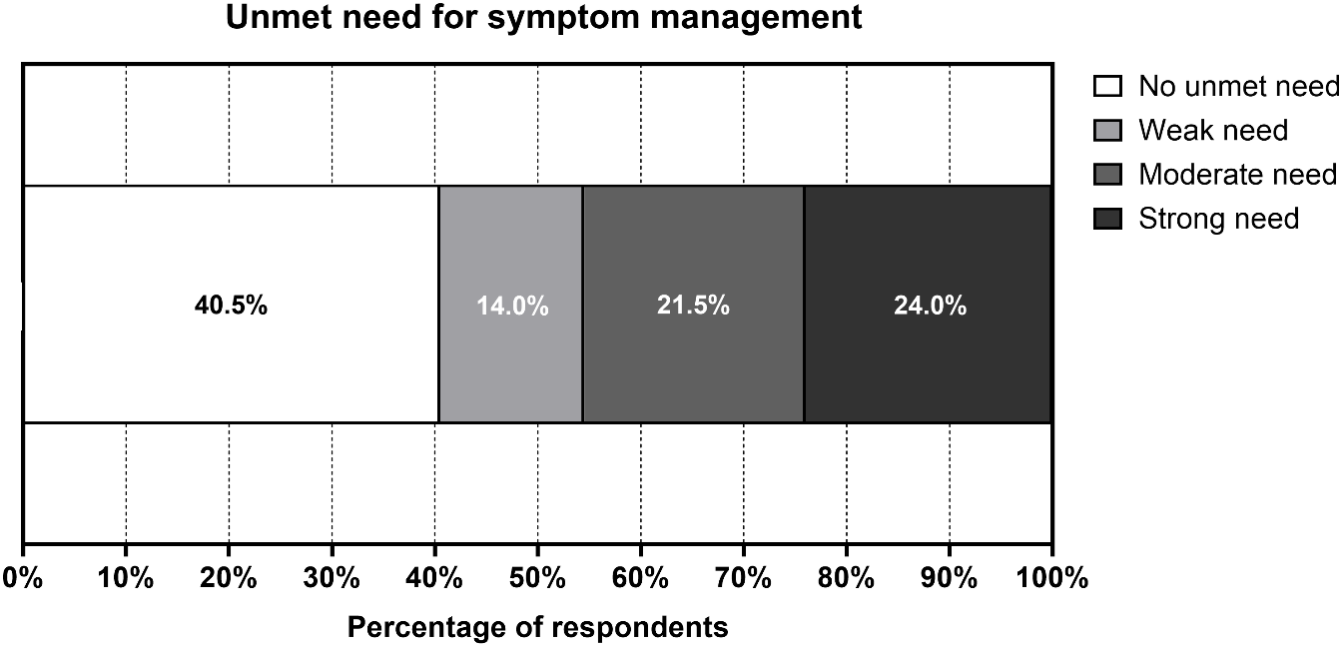
Unmet Need for Symptom Management (N = 200). Figures were constructed using GraphPad Prism version 9 (GraphPad Software, San Diego, USA). Nearly 60% of participants indicated an unmet need for symptom management, while around one-fourth of them reported a strong unmet need.

There were 119 (59.5%) participants who indicated an unmet need for symptom management, with 24.0% reporting a strong need (**Figure 2**). Univariate logistic regression revealed that the unmet need for symptom management decreased with post-treatment duration (crude odds ratio [OR_crude_] = 0.983, 95% CI = 0.974 – 0.992, p < 0.001), and age (OR_crude_ = 0.965, 95% CI = 0.939 – 0.992, p = 0.012). No associations were identified with other characteristics (**Table 5**).

**Table 5:** Association between unmet needs for symptom management and patients’ characteristics.

Categorical variable	Frequency	OR_crude_ (95% CI)
Gender		
Male	148	1
Female	52	0.904 (0.476 - 1.716)
Educational level		
Not at all	5	1
Primary school	123	0.330 (0.036 - 3.041)
Secondary school	33	0.438 (0.044 - 4.378)
Diploma and Bachelor’s degree	26	0.400 (0.039 - 4.109)
Master’s degree or above	13	0.400 (0.034 - 4.681)
Employment status		
Full-time job	81	1
Part-time job	19	1.343 (0.463 - 3.901)
Not employed	14	0.620 (0.198 - 1.937)
Retired	74	0.814 (0.428 - 1.546)
Others	12	0.868 (0.253 - 2.976)
Marital status		
Not married	40	1
Married	160	1.430 (0.712 - 2.872)
Living status		
Living alone	10	1
Living with spouse	52	2.722 (0.633 - 11.701)
Living with family members	132	3.952 (0.977 - 15.993)
Others	6	11.667 (0.922 - 147.563)
Received chemotherapy		
No	4	1
Yes	151	1.271 (0.663 - 2.436)
T staging		
1	94	1
2	22	2.207 (0.750 - 6.496)
3	65	0.712 (0.376 - 1.349)
4	19	0.893 (0.328 - 2.427)
N staging		
0	55	1
1	56	0.957 (0.448 - 2.041)
2	62	0.759 (0.364 - 1.582)
3	26	1.810 (0.652 - 5.022)
M staging		
0	196	1
1	3	0.331 (0.029 - 3.707)
TNM staging		
1	39	1
2	35	1.308 (0.514 - 3.325)
3	84	0.982 (0.456 - 2.111)
4	42	1.545 (0.628 - 3.806)
Number of chronic illnesses		
0	10	1
1	52	0.567 (0.296 - 1.090)
2 or above	132	1.426 (0.600 - 3.391)
Smoker		
No	184	1
Yes	16	3.189 (0.879 - 11.572)
Drinker		
No	144	1
Yes	56	0.968 (0.516 - 1.813)
Continuous variable	Mean ± SD	OR_crude_ (95% CI)
Post-treatment time (Month)	52.6 ± 33.6	0.983 (0.974 - 0.992)^‡^
Age	58.2 ± 10.9	0.965 (0.939 - 0.992)^*^
Age at diagnosis	53.4 ± 10.8	0.979 (0.953 - 1.006)

95% CI, 95% confidence interval; OR_crude_, crude odds ratio.

^*^p<0.05.

^‡^p<0.001.

Univariate logistics regression analysis demonstrated that the unmet need for symptom management increased with general, nutrition impact symptoms, social interaction impact symptoms and interference items, as well as the MeanTop5 symptoms in an independent manner (**Table 6**). No association was found with the gastrointestinal symptoms items.

**Table 6:** Association between unmet needs for symptom management and MDASI subscales.

MDASI subscale	Mean ± SD	OR_crude_ (95% CI)
General	2.41 ± 1.82	1.523 (1.261 - 1.840)^‡^
Gastrointestinal	0.58 ± 1.51	1.149 (0.927 - 1.424)
Nutrition impact	2.75 ± 2.01	1.356 (1.150 - 1.598)^‡^
Social interaction impact	1.54 ± 1.83	1.235 (1.037 - 1.470)^*^
Interference	1.82 ± 2.11	1.374 (1.161 - 1.628)^‡^
MeanTop5	3.81 ± 2.25	1.351 (1.171 - 1.558)^‡^

95% CI, 95% confidence interval; OR_crude_, crude odds ratio.

^*^p<0.05.

^‡^p<0.001.

A multiple logistics regression analysis revealed that the unmet need for symptom management increased with general symptoms (adjusted odds ratio [OR_adj_] = 1.566, 95% CI = 1.282 – 1.914, p < 0.001), but decreased with the post-RT month (OR_adj_ = 0.981, 95% CI = 0.972 – 0.991, p < 0.001). An alternative approach was analyzed using multiple logistic regression, in which the unmet need for symptom management increased with symptom burden represented by MeanTop5 (OR_adj_ = 1.379, 95% CI = 1.185 – 1.604, p < 0.001), but decreased with the post-RT month (OR_adj_ = 0.981, 95% CI = 0.972 – 0.991, p < 0.001) (**Table 7**).

**Table 7:** Multiple logistic regression of the unmet need for symptom management.

Four-factor model	Mean ± SD	OR_adj_ (95% CI)
Post-treatment time (month)	52.6 ± 33.6	0.981 (0.972 - 0.991)^‡^
MDASI-H&N General items	2.41 ± 1.82	1.566 (1.282 - 1.914)^‡^
MeanTop5 model	Mean ± SD	OR_adj_ (95% CI)
Post-treatment time (month)	52.6 ± 33.6	0.981 (0.972 - 0.991)^‡^
MeanTop5	3.81 ± 2.25	1.379 (1.185 - 1.604)^‡^

95% CI, 95% confidence interval; OR_adj_, adjusted odds ratio; SD, standard deviation.

^‡^p<0.001.

### 3.4 Additional symptoms and concerns

Auditory-related problems were raised as significant post-RT symptoms in the open-ended responses, which included tinnitus, hearing impairment, middle ear effusion and tympanic membrane perforation. Neck stiffness and pain were also mentioned as additional concerns which were not included in the MDASI scale.

## 4 Discussion

Late radiation side effects remain a significant concern during cancer survivorship despite the advancements in clinical oncology in the past few decades. This study presented a comprehensive survey on patient-reported symptoms, HRQoL and unmet need for symptom management among local NPC survivors. Several oral and upper gastrointestinal symptoms, including dry mouth, mucus problems, difficulty swallowing or chewing, and problems with teeth or gums, were listed as top symptoms, and a similar result was observed in an NPC survivors’ survey in Canada (5). A negative correlation between the severity of symptoms and post-treatment duration was found in nausea, lack of appetite and dysgeusia. However, these associations were weak, and no significant differences were identified across various post-treatment time intervals. This suggests a varied manifestation and evolution of late symptoms throughout the follow-up period.

Xerostomia has also been reported as a prevalent clinician-reported side effect (48.7% - 79.1%) in Chinese NPC survivors (3, 4). It was well noted that NPC survivors suffered from impaired oral intake, which could result in poor nutrition and diminished HRQoL (2). Irradiation to the salivary glands induced atrophy of acinar cells and fibrosis, leading to reduced salivary production. As saliva plays a role in bolus formation and lubrication during swallowing, reduced production could significantly impair ingestion in conjunction with damaged swallowing muscles due to radiation. Furthermore, saliva contains a wide range of defense proteins, such as immunoglobulins and lysozyme. Hyposalivation could lead to thick mucus in the mouth and throat and increase the risks of oral infection and tooth decay. These symptoms interact with each other and complicate the oral conditions among NPC survivors (19). Although some studies suggested considerable recovery of saliva production after completion of RT, this remains a major oral symptom and health challenge which impairs survivors’ oral health and quality of life (20, 21). Our findings highlighted the persistent suffering of NPC survivors from oral symptoms, regardless of the time after RT completion. Consequently, continuous supportive care is essential, encompassing routine standard assessments, regular dental follow-ups, and active management to alleviate side effects and support oral intake.

Dysmnesia, referring to problems in memory, was also listed as one of the top five symptoms in our study. Temporal lobe injury (TLI) was a well-known sequela of NPC RT, which could lead to cognitive and neurobehavioral impairments. McDowell et al. (22) revealed long-term cognitive and neurobehavioral impairments including dysmnesia, apathy, disinhibition and executive dysfunction, which were associated with a radiation dose of >75Gy to the temporal lobe. Feng et al. (23) also demonstrated that a dose delivered to 2cc of the temporal lobe was an independent dose predictor of TLI. Although magnetic resonance imaging (MRI) is a key tool for assessing TLI, it’s important to note that clinical symptoms might not always align with MRI findings, owing to the brain’s complexity and co-morbidities (24). Patient-reported outcomes may reveal unique aspects of symptom burden that are potentially underrated by clinicians.

The MDASI-HN is a validated PRO tool to evaluate symptoms in H&N cancer patients. Cleeland et al. (11) and Rosenthal et al. (25) conducted initial validation on the MDASI core and the MDASI-HN scales, revealing a two-factor model for both core and HN-specific subscales respectively among H&N cancer patients. Xiao et al. (15) further validated the MDASI-HN-C among Chinese NPC survivors, demonstrating similar results except for the components of each factor in the HN-specific subscales. However, some acute side effects subside and recover with time, while some late symptoms emerge months or years after treatment completion, leading to a unique side effect profile of NPC survivors when compared with patients receiving active treatment (26). For instance, neck pain and stiffness, tinnitus and other hearing problems were raised as significant late-presenting symptoms among NPC survivors in our cohort, which were also well-documented in other studies (5, 19, 27). Other side effects, including trismus and osteoradionecrosis, have been documented in the later stages of survivorship. These symptoms were not included in the first item generation stage of the initial development of the MDASI, in which patients were recruited before, during, or after surgery, chemotherapy, or RT (25). The difference between sampling populations could alter the applicability of the tool in the symptom burden assessment of patients or survivors. Incorporating the previously mentioned symptoms could more effectively address the unique symptom pattern seen among NPC survivors. Modifying and validating the inventory would aid in accurately assessing the symptom burden for specific cancer types in the H&N region.

To the best of our knowledge, this is the first study investigating the factors associated with the unique unmet need for symptom management among NPC survivors. An unmet need refers to the perceived insufficiency of a certain aspect of life (28). Living with or recovering from cancer can significantly affect various aspects of life, often leading to diverse unmet needs that urgently require attention (29). Perceptions of needs can vary among individuals with similar clinical conditions, as they are shaped by the complex interplay between personal demands and the assistance available to them. The CASUN scale is designed to assess the unmet needs of cancer survivors under five distinct domains. An unmet need for symptom management was an integral item under the ‘Quality of Life’ factor (30, 31). An unmet need for symptom management in cancer survivors suggests a necessity for additional support to manage treatment-induced toxicity. It has been recommended that regular assessment of symptom burden, advancement in symptom management, improved availability and accessibility of service, timely referral and continuous support of psychosocial aspects are crucial in cancer survivorship care (27, 32).

Our findings suggest that higher symptom severity, as indicated by the general items or the MeanTop5 scale, intuitively correlates with a greater unmet need for symptom management. Intriguingly, we observed a negative association between the post-RT duration and the unmet need for symptom management, despite the symptom burden showing no significant variation over time. This echoed the complexity of survivors’ perceptions of needs and resilience to adapt to their new ‘normal’ after recovering from the illness. Previous studies have shown a similar trend in the total number of unmet needs during survivorship, with younger age and earlier phases of survivorship being significant predictors of a higher number of needs (33, 34). While the severity of symptoms could vary among survivors irrespective of the post-RT duration, a personalized supportive care plan, based on assessing and stratifying symptom burden into different levels, could effectively address patients’ concerns about post-treatment side effects.

There were several limitations in this study. First, we recruited survivors attending the follow-up clinic in a single center using convenience sampling, which could result in a biased sample with limited generalizability. Second, survivors with severe hearing impairments or those who cannot follow commands were excluded from the survey. This may represent a group of severely impacted survivors, in which our study may underrate the overall symptom burden of this cohort. Additionally, survivors treated over ten years ago were excluded, potentially leading to an underestimation of very late toxicities, such as extensive fibrotic changes. Also, common auditory issues and neck fibrosis symptoms were not covered in the current version of the MDASI-HN-C.

## 5 Conclusions

Almost all NPC survivors experienced post-treatment symptoms, regardless of the time elapsed since radiation therapy. Common issues included upper gastrointestinal symptoms and memory impairment. The MDASI-HN-C tool effectively assessed NPC survivors’ symptom burden and its association with the unmet need for symptom management, which diminished over the months following RT. Further investigations on exploring the complex interplay between symptom severity and survivor perception of needs are warranted. Implementing personalized supportive care strategies, complete with regular assessments and stratification systems, is crucial for strengthening cancer survivorship care.

## Data availability statement

The original contributions presented in the study are included in the article/**Supplementary Material**. Further inquiries can be directed to the corresponding authors.

## Ethics statement

The studies involving humans were approved by the Institutional Review Board of The Hong Kong Polytechnic University and the Clinical Research Ethics Committee of the Joint Chinese University of Hong Kong-New Territories East Cluster. The studies were conducted in accordance with the local legislation and institutional requirements. The participants provided their written informed consent to participate in this study.

## Author contributions

VT: Conceptualization, Data curation, Formal analysis, Investigation, Methodology, Project administration, Resources, Validation, Visualization, Writing – original draft, Writing – review & editing. JC: Conceptualization, Data curation, Investigation, Methodology, Project administration, Resources, Writing – review & editing. SY: Conceptualization, Data curation, Investigation, Methodology, Project administration, Resources, Writing – review & editing. VK: Conceptualization, Data curation, Project administration, Resources, Writing – review & editing. CC: Conceptualization, Data curation, Resources, Software, Writing – review & editing. KW: Conceptualization, Data curation, Project administration, Resources, Supervision, Writing – review & editing. SL: Conceptualization, Data curation, Funding acquisition, Methodology, Resources, Supervision, Writing – review & editing.
