# Supplementary material for: Examining patient-reported late toxicity and its association with quality of life and unmet need for symptom management among nasopharyngeal cancer survivors: a cross-sectional survey
Source: Front Oncol. 2024 Apr 17;14:1378973. doi: 10.3389/fonc.2024.1378973 (PMC11061844; doi:10.3389/fonc.2024.1378973)
Supplement: Supplementary file 1 [file DataSheet_1.pdf]

Appendix 1. Sample questionnaire_ENGLISH

Date of submission:____________ Date of RT completion (MM/YYYY)：________________________________

Examining Patient-Reported Late Toxicity and Its Association with Quality of Life and Unmet Need for Symptom Management among Nasopharyngeal Cancer Survivors: A Cross-Sectional Survey

1. Demographics

1.1 Gender

□Male □Female

1.2 Age __________________________

1.3 Educational level

□Not at all □Primary school □Secondary school □Diploma and Bachelor's degree

□Master's degree or above □Others; please specify:____________________

1.4 Employment status

□Full-time job □Part-time job □Sick leave □Not employed □Retried □Housewife □Student

□Others; please specify:___________________

1.5 Marital status

□Single □In a relationship □Married □Divorced □Widowed □Not to mention

□Others; please specify:___________________

1.6 Living status

□Living alone □Living with spouse □Living with family members □Living with others

□Not to mention □Others; please specify:___________________

1.7 Are you suffering from chronic disease?

□Yes (Please tick all applicable choices) □No

**Heart disease**	□	**Diabetes mellitus**	□	**Stroke**	□	**Asthma**	□
**Hyper-choles-terolemia**	□	**Kidney disease**	□	**Hyper-tension**	□	**Chronic Pulmo-nary Disease**	□
Others; please specify:___________________							

1.8 Have you received chemotherapy?

□Yes □No □Not sure

1.9 Have you received immunotherapy?

□Yes □No □Not sure

1.10 Have you received other cancer treatment?

□Yes; please specify:____________________ □No □Not sure

1.11 Disease staging

□I □II □III □IV □Not sure

2. M. D. Anderson Symptom Inventory - Head & Neck

Part I. How severe are your symptoms?

People with cancer frequently have symptoms that are caused by their disease or by their treatment. We ask you to rate how severe the following symptoms have been ***in the last 24 hours***.

Please circle below from 0 (symptom has not been present) to 10 (the symptom was as bad as you can imagine it could be) for each item.

		**NOT PRESENT**										**AS BAD AS YOU CAN IMAGINE**
2.1	Your **pain** at its WORST?	0	1	2	3	4	5	6	7	8	9	10
2.2	Your **fatigue (tiredness)** at its WORST?	0	1	2	3	4	5	6	7	8	9	10
2.3	Your **nausea** at its WORST?	0	1	2	3	4	5	6	7	8	9	10
2.4	Your **disturbed sleep** at its WORST?	0	1	2	3	4	5	6	7	8	9	10
2.5	Your feeling of being **distressed (upset)** at its WORST?	0	1	2	3	4	5	6	7	8	9	10
2.6	Your **shortness of breath** at its WORST?	0	1	2	3	4	5	6	7	8	9	10
2.7	Your problem with **remembering things** at its WORST?	0	1	2	3	4	5	6	7	8	9	10
2.8	Your problem with **lack of appetite** at its WORST?	0	1	2	3	4	5	6	7	8	9	10
2.9	Your feeling **drowsy (sleepy)** at its WORST?	0	1	2	3	4	5	6	7	8	9	10

2.10	Your having a **dry mouth** at its WORST?	0	1	2	3	4	5	6	7	8	9	10
2.11	Your feeling **sad** at its WORST?	0	1	2	3	4	5	6	7	8	9	10
2.12	Your **vomiting** at its WORST?	0	1	2	3	4	5	6	7	8	9	10
2.13	Your **numbness or tingling** at its WORST?	0	1	2	3	4	5	6	7	8	9	10
2.14	Your problem with **mucus** in your mouth and throat at its WORST?	0	1	2	3	4	5	6	7	8	9	10
2.15	Your difficulty **swallowing/ chewing** at its WORST?	0	1	2	3	4	5	6	7	8	9	10
2.16	Your **choking/coughing** (food/ liquids going down the wrong pipe) at its WORST?	0	1	2	3	4	5	6	7	8	9	10
2.17	Your difficulty with **voice/speech** at its WORST?	0	1	2	3	4	5	6	7	8	9	10
2.18	Your **skin pain/burning/rash** at its WORST?	0	1	2	3	4	5	6	7	8	9	10
2.19	Your **constipation** at its WORST?	0	1	2	3	4	5	6	7	8	9	10
2.20	Your problem with **tasting food** at its WORST?	0	1	2	3	4	5	6	7	8	9	10
2.21	Your **mouth/throat** sores at their WORST?	0	1	2	3	4	5	6	7	8	9	10
2.22	Your problem with your **teeth or gums** at its WORST?	0	1	2	3	4	5	6	7	8	9	10

Part II. How have your symptoms interfered with your life?

Symptoms frequently interfere with how we feel and function. How much have your symptoms interfered with the following items in the ***last 24 hours***?

Please circle below from 0 (did not interfere) to 10 (interfered completely) for each item.

		**Did not interfere**										**Interfered completely**
2.23	**General activity?**	0	1	2	3	4	5	6	7	8	9	10
2.24	**Mood?**	0	1	2	3	4	5	6	7	8	9	10
2.25	**Work (including work around the house)?**	0	1	2	3	4	5	6	7	8	9	10
2.26	**Relations with other people?**	0	1	2	3	4	5	6	7	8	9	10
2.27	**Walking?**	0	1	2	3	4	5	6	7	8	9	10
2.28	**Enjoyment of life?**	0	1	2	3	4	5	6	7	8	9	10

3. Quality of Life (FACT-HN)

Below is a list of statements that other people with your illness have said are important. **Please circle or mark one number per line to indicate your response as it applies to the *past 7 days.***

	**PHYSICAL WELL-BEING**	Not at all	A little bit	Some-what	Quite a bit	Very much
3.1	I have a lack of energy	0	1	2	3	4
3.2	I have nausea	0	1	2	3	4
3.3	Because of my physical condition, I have trouble meeting the needs of my family	0	1	2	3	4
3.4	I have pain	0	1	2	3	4
3.5	I am bothered by side effects of treatment	0	1	2	3	4
3.6	I feel ill	0	1	2	3	4
3.7	I am forced to spend time in bed	0	1	2	3	4

	**SOCIAL/FAMILY WELL-BEING**	Not at all	A little bit	Some-what	Quite a bit	Very much
3.8	I feel close to my friends	0	1	2	3	4
3.9	I get emotional support from my family	0	1	2	3	4
3.10	I get support from my friends	0	1	2	3	4
3.11	My family has accepted my illness	0	1	2	3	4
3.12	I am satisfied with family communication about my illness	0	1	2	3	4
3.13	I feel close to my partner (or the person who is my main support)	0	1	2	3	4
3.14	I am satisfied with my sex life	0	1	2	3	4

	**EMOTIONAL WELL-BEING**	Not at all	A little bit	Some-what	Quite a bit	Very much
3.15	I feel sad	0	1	2	3	4
3.16	I am satisfied with how I am coping with my illness	0	1	2	3	4

3.17	I am losing hope in the fight against my illness	0	1	2	3	4
3.18	I feel nervous	0	1	2	3	4
3.19	I worry about dying	0	1	2	3	4
3.20	I worry that my condition will get worse	0	1	2	3	4

	**FUNCTIONAL WELL-BEING**	Not at all	A little bit	Some-what	Quite a bit	Very much
3.21	I am able to work (include work at home)	0	1	2	3	4
3.22	My work (include work at home) is fulfilling	0	1	2	3	4
3.23	I am able to enjoy life	0	1	2	3	4
3.24	I have accepted my illness	0	1	2	3	4
3.25	I am sleeping well	0	1	2	3	4
3.26	I am enjoying the things I usually do for fun	0	1	2	3	4
3.27	I am content with the quality of my life right now	0	1	2	3	4

	**ADDITIONAL CONCERNS**	Not at all	A little bit	Some-what	Quite a bit	Very much
3.28	I am able to eat the foods that I like	0	1	2	3	4
3.29	My mouth is dry	0	1	2	3	4
3.30	I have trouble breathing	0	1	2	3	4
3.31	My voice has its usual quality and strength	0	1	2	3	4
3.32	I am able to eat as much food as I want	0	1	2	3	4
3.33	I am unhappy with how my face and neck look	0	1	2	3	4
3.34	I can swallow naturally and easily	0	1	2	3	4
3.35	I smoke cigarettes or other tobacco products	0	1	2	3	4
3.36	I drink alcohol (e.g. beer, wine, etc.)	0	1	2	3	4
3.37	I am able to communicate with others	0	1	2	3	4

3.38	I can eat solid foods	0	1	2	3	4
3.39	I have pain in my mouth, throat or neck	0	1	2	3	4

4. Cancer Survivors' Unmet Needs

Please read the following item and tick (✓) the answer that best describes your experience. There are no right or wrong answers.

	In the last month	**No need**	**Have need, but need is being met**	**Weak**	**Moderate**	**Strong**
4.1	I need help to manage ongoing side effects and/or complications of treatment…					

~End of the questionnaire~
