# Supplementary material for: Examining patient-reported late toxicity and its association with quality of life and unmet need for symptom management among nasopharyngeal cancer survivors: a cross-sectional survey
Source: Front Oncol. 2024 Apr 17;14:1378973. doi: 10.3389/fonc.2024.1378973 (PMC11061844; doi:10.3389/fonc.2024.1378973)
Supplement: Supplementary file 2 [file Table_1.docx]

## Supplementary table 1. Association between MDASI subscales and patients’ characteristics

|  | | | MDASI subscales | | | | | |
| --- | --- | --- | --- | --- | --- | --- | --- | --- |
|  |  |  | General | Gastrointestinal | Nutrition impact | Social interaction impact | Interference | MeanTop5 |
| *Categorical variables* | | n | Beta coefficient (95% CI) | | | | | |
| Gender | |  |  |  |  |  |  |  |
|  | Male | 148 | 1 | 1 | 1 | 1 | 1 | 1 |
|  | Female | 52 | 0.129 (-0.444 - 0.701) | 0.120 (-0.356 - 0.595) | 0.441 (-0.191 - 1.072) | -0.123 (-0.700 - 0.454) | 0.252 (-0.412 - 0.915) | 0.397 (-0.309 - 1.104) |
| Educational level | |  |  |  |  |  |  |  |
|  | Not at all | 5 | 1 | 1 | 1 | 1 | 1 | 1 |
|  | Primary school | 123 | 1.175 (-0.403 - 2.753) | 0.598 (-0.734 - 1.929) | 1.984 (0.220 - 3.748)^*^ | 0.766 (-0.855 - 2.388) | 0.350 (-1.511 - 2.212) | 2.228 (0.259 - 4.197)^*^ |
|  | Secondary school | 33 | 0.485 (-1.175 - 2.145) | 0.227 (-1.174 - 1.628) | 1.360 (-0.496 - 3.215) | 0.402 (-1.303 - 2.108) | -0.024 (-1.982 - 1.934) | 1.553 (-0.519 - 3.624) |
|  | Diploma and Bachelor’s degree | 26 | 1.811 (0.122 - 3.500)^*^ | 0.904 (-0.522 - 2.329) | 1.938 (0.050 - 3.827)^*^ | 1.008 (-0.728 - 2.743) | 0.868 (-1.125 - 2.860) | 2.403 (0.295 - 4.511)^*^ |
|  | Master’s degree or above | 13 | 1.524 (-0.295 - 3.344) | 0.808 (-0.728 - 2.344) | 1.862 (-0.173 - 3.896) | 1.097 (-0.773 - 2.968) | 0.964 (-1.183 - 3.111) | 2.265 (-0.007 - 4.536) |
| Employment status | |  |  |  |  |  |  |  |
|  | Full-time job | 81 | 1 | 1 | 1 | 1 | 1 | 1 |
|  | Part-time job | 19 | 0.774 (-0.120 - 1.688) | 0.287 (-0.461 - 1.034) | 0.324 (-0.666 - 1.315) | 0.196 (-0.714 - 1.107) | 0.660 (-0.376 - 1.696) | 0.605 (-0.502 - 1.712) |
|  | Not employed | 14 | 0.332 (-0.683 - 1.347) | -0.268 (-1.116 - 0.581) | 0.534 (-0.591 - 1.658) | -0.191 (-1.225 - 0.843) | 0.836 (-0.340 - 2.013) | 0.533 (-0.725 - 1.790) |
|  | Retired | 74 | -0.029 (-0.593 - 0.535) | -0.215 (-0.686 - 0.257) | 0.121 (-0.504 - 0.746) | 0.178 (-0.397 - 0.753) | 0.164 (-0.490 - 0.817) | 0.201 (-0.498 - 0.899) |
|  | Others | 12 | 0.759 ( -0.326 - 1.845) | -0.244 (-1.151 - 0.663) | 1.272 (0.069 - 2.474)^*^ | 0.270 (-0.836 - 1.375) | 1.168 (-0.089 - 2.425) | 1.397 (0.053 - 2.741)^*^ |
| Marital status | |  |  |  |  |  |  |  |
|  | Not married | 40 | 1 | 1 | 1 | 1 | 1 | 1 |
|  | Married | 160 | 0.572 (-0.051 - 1.195) | 0.016 (-0.506 - 0.537) | 0.308 (-0.386 - 1.002) | 0.508 (-0.121 - 1.137) | 0.279 (-0.448 - 1.007) | 0.564 (-0.210 - 1.337) |
| Living status | |  |  |  |  |  |  |  |
|  | Living alone | 10 | 1 | 1 | 1 | 1 | 1 | 1 |
|  | Living with spouse | 52 | 1.426 (0.224 - 2.628)^*^ | 0.362 (-0.648 - 1.372) | 0.691 (-0.663 - 2.045) | 1.635 (0.422 - 2.847)^†^ | 0.658 (-0.760 - 2.077) | 1.050 (-0.457 - 2.557) |
|  | Living with family members | 132 | 1.637 (0.495 - 2.779)^†^ | 0.517 (-0.442 - 1.477) | 0.775 (-0.511 - 2.060) | 1.601 (0.449 - 2.753)^†^ | 0.747 (-0.600 - 2.094) | 1.229 (-0.203 - 2.660) |
|  | Others | 6 | 1.058 (-0.740 - 2.855) | 1.317 (-0.194 - 2.827) | 0.883 (-1.141 - 2.908) | 1.944 (0.131 - 3.758)^*^ | 0.761 (-1.360 - 2.882) | 0.733 (-1.520 - 2.987) |
| Received chemotherapy | |  |  |  |  |  |  |  |
|  | No | 4 | 1 | 1 | 1 | 1 | 1 | 1 |
|  | Yes | 151 | 0.255 (-0.328 - 0.838) | 0.289 (-0.195 - 0.772) | 0.616 (-0.025 - 1.257) | 0.283 (-0.305 - 0.870) | 0.550 (-0.123 - 1.223) | 0.554 (-0.165 - 1.273) |
| T staging | |  |  |  |  |  |  |  |
|  | 1 | 94 | 1 | 1 | 1 | 1 | 1 | 1 |
|  | 2 | 22 | 0.858 (0.030 - 1.687)^*^ | 0.753 (0.068 - 1.439)^*^ | 1.338 (0.427 - 2.248) | 0.741 (-0.099 - 1.582) | 0.986 (0.021 - 1.951)^*^ | 1.443 (0.426 - 2.460)^†^ |
|  | 3 | 65 | 0.369 (-0.195 - 0.933) | 0.409 (-0.057 - 0.876) | 0.521 (-0.099 - 1.142) | 0.389 (-0.184 - 0.961) | 0.424 (-0.233 - 1.081) | 0.396 (-0.296 - 1.089) |
|  | 4 | 19 | -0.343 (-1.223 - 0.536) | -0.251 (-0.979 - 0.476) | 0.073 (-0.894 - 1.040) | 0.137 (-0.755 - 1.029) | 0.219 (-0.806 - 1.243) | -0.410 (-1.490 - 0.670) |
| N staging | |  |  |  |  |  |  |  |
|  | 0 | 55 | 1 | 1 | 1 | 1 | 1 | 1 |
|  | 1 | 56 | 0.013 (-0.658 - 0.684) | 0.237 (-0.305 - 0.778) | 0.101 (-0.639 - 0.841) | -0.085 (-0.760 - 0.591) | 0.298 (-0.477 - 1.073) | -0.051 (-0.882 - 0.781) |
|  | 2 | 62 | 0.295 (-0.359 - 0.950) | 0.633 (0.104 - 1.161)^*^ | 0.619 (-0.102 - 1.341) | 0.395 (-0.264 - 1.054) | 0.477 (-0.280 - 1.233) | 0.504 (-0.307 - 1.315) |
|  | 3 | 26 | 0.281 (-0.560 - 1.122) | 0.265 (-0.414 - 0.943) | 0.023 (-0.904 - 0.951) | 0.184 (-0.663 - 1.030) | 0.619 (-0.353 - 1.591) | 0.049 (-0.993 - 1.092) |
| M staging | |  |  |  |  |  |  |  |
|  | 0 | 196 | 1 | 1 | 1 | 1 | 1 | 1 |
|  | 1 | 3 | -1.493 (-3.555 - 0.569) | -0.587 (-2.304 - 1.131) | -1.037 (-3.324 - 1.250) | -0.777 (-2.862 - 1.309) | 0.243 (-2.157 - 2.642) | -1.349 (-3.900 - 1.203) |
| TNM staging | |  |  |  |  |  |  |  |
|  | 1 | 39 | 1 | 1 | 1 | 1 | 1 | 1 |
|  | 2 | 35 | 0.408 (-0.415 - 1.231) | 0.525 (-0.153 - 1.203) | 0.459 (-0.446 - 1.365) | -0.144 (-0.974 - 0.687) | 0.573 (-0.378 - 1.523) | 0.512 (-0.502 - 1.526) |
|  | 3 | 84 | 0.454 (-0.231 - 1.139) | 0.556 (-0.009 - 1.120) | 0.794 (0.041 - 1.548)^*^ | 0.304 (-0.387 - 0.995) | 0.716 (-0.076 - 1.507) | 0.735 (-0.109 - 1.579) |
|  | 4 | 42 | 0.160 (-0.626 - 0.946) | 0.151 (-0.497 - 0.799) | 0.322 (-0.543 - 1.187) | 0.129 (-0.663 - 0.922) | 0.756 (-0.152 - 1.664) | 0.135 (-0.834 - 1.103) |
| Number of chronic illnesses # | |  |  |  |  |  |  |  |
|  | 0 | 10 | 1 | 1 | 1 | 1 | 1 | 1 |
|  | 1 | 52 | 0.376 (-0.207 - 0.959) | 0.043 (-0.443 - 0.528) | 0.016 (-0.632 - 0.664) | 0.015 (-0.572 - 0.602) | 0.205 (-0.473 - 0.883) | 0.264 (-0.459 - 0.987) |
|  | 2 or above | 132 | 0.264 (-0.460 - 0.989) | -0.094 (-0.698 - 0.510) | 0.102 (-0.704 - 0.908) | 0.546 (-0.183 - 1.275) | 0.211 (-0.631 - 1.054) | 0.289 (-0.611 - 1.188) |
| Smoker | |  |  |  |  |  |  |  |
|  | No | 184 | 1 | 1 | 1 | 1 | 1 | 1 |
|  | Yes | 16 | 0.468 (-0.455 - 1.392) | 0.156 (-0.612 - 0.925) | 1.267 (0.257 - 2.277)^*^ | 0.160 (-0.773 - 1.093) | 0.818 (-0.250 - 1.886) | 0.917 (-0.222 - 2.057) |
| Drinker | |  |  |  |  |  |  |  |
|  | No | 144 | 1 | 1 | 1 | 1 | 1 | 1 |
|  | Yes | 56 | 0.075 (-0.484 - 0.635) | -0.117 (-0.581 - 0.348) | 0.115 (-0.505 - 0.734) | 0.259 (-0.304 - 0.821) | -0.138 (-0.786 - 0.511) | 0.121 (-0.572 - 0.813) |
| *Continuous variables* | | Mean + SD | Unstandardised Beta coefficient (95% CI) | | | | | |
|  | Post-treatment time (Month) | 52.6 + 33.6 | -0.002 (-0.009 - 0.006) | -0.002 (-0.008 - 0.005) | -0.004 (-0.012 - 0.005) | 0.000 (-0.008 - 0.008) | -0.007 (-0.016 - 0.001) | -0.002 (-0.011 - 0.008) |
|  | Age | 58.2 + 10.9 | -0.023 (-0.046 - 0.000) | -0.016 (-0.035 - 0.003) | -0.021 (-0.047 - 0.004) | -0.007 (-0.031 - 0.016) | -0.021 (-0.048 - 0.006) | -0.021 (-0.050 - 0.008) |
|  | Age at diagnosis | 53.4 + 10.8 | -0.022 (-0.045 - 0.002) | -0.015 (-0.034 - 0.005) | -0.019 (-0.045 - 0.007) | -0.008 (-0.031 - 0.016) | -0.016 (-0.043 - 0.012) | -0.019 (-0.049 - 0.010) |

*95% CI*, 95% confidence interval

^*^ p<0.05

^†^ p<0.01

^‡^ p<0.001
